# The geriatric nutritional risk index predicts short-term mortality in older patients with urosepsis: a retrospective cohort study with external validation

**DOI:** 10.3389/fnut.2026.1793046

**Published:** 2026-07-02

**Authors:** Lihua Zhu, Zirong Gao, Ying Yu, Zhen Wu, Yafang Diao, Junhua Zhang, Xiangqian Nie, Cheng Zha

**Affiliations:** 1 Zhejiang Provincial People's Hospital Bijie Hospital (The First People’s Hospital of Bijie), Bijie, China; 2 Department of Neuro Intensive Care Unit, The Affiliated Jinyang Hospital of Guizhou Medical University, Guiyang, China

**Keywords:** elderly nutrition risk index, MIMIC-IV database, risk prediction, short term mortality rate, urinary sepsis

## Abstract

**Background:**

Urosepsis, a severe complication of urinary tract infection, is associated with high mortality, especially in older adults. The Geriatric Nutritional Risk Index (GNRI) is a simple nutritional assessment tool, but its prognostic value specifically in urosepsis remains underexplored.

**Methods:**

This retrospective cohort study included 1,218 patients aged ≥65 years with urosepsis from the MIMIC-IV database (derivation cohort) and 178 patients from an external hospital (validation cohort). Nutritional risk was categorized using GNRI. Cox proportional hazards models were used to assess the association between GNRI and 28-day ICU/in-hospital mortality. The incremental predictive value of GNRI added to established severity scores was evaluated using ROC analysis.

**Results:**

A total of 1,218 urosepsis patients were included. The 28-day ICU mortality and in-hospital mortality rates were 18.7 and 17.2%. After multivariable adjustment, lower GNRI was independently associated with increased 28-day ICU mortality (adjusted HR = 0.96, 95% CI: 0.94–0.98, *p* < 0.001) and in-hospital mortality (adjusted HR = 0.96, 95% CI: 0.94–0.98, *p* < 0.001). Patients in the high GNRI-risk group had significantly higher mortality compared to the no-risk group (ICU mortality: adjusted HR = 2.34, 95% CI: 1.07–5.11, *p* = 0.034; in-hospital mortality: adjusted HR = 2.63, 95% CI: 1.05–6.58, *p* = 0.039). Adding GNRI significantly improved the predictive accuracy of all six severity scores (e.g., SOFA AUC increased from 0.625 to 0.709, *p* < 0.001). Subgroup analyses showed consistent trends across most demographics and comorbidities. These findings were validated in the external cohort, where a higher GNRI remained protective against 28-day mortality (adjusted HR = 0.95, 95% CI: 0.91–1.00, *p* = 0.033).

**Conclusion:**

GNRI is an independent predictor of short-term mortality in older patients with urosepsis and enhances the prognostic accuracy of existing severity scores. Early nutritional assessment and intervention may improve outcomes in this high-risk population.

## Background

Sepsis syndrome is a complex host inflammatory response to infection, associated with high mortality and recognized as a leading cause of death in non-cardiac intensive care units (1, 2). Infections originating from different anatomical sites can trigger this condition, with urogenital tract infections accounting for 9–31% of all sepsis cases. These cases—termed urosepsis—represent a severe complication of urinary tract infections (UTIs) and pose a substantial threat to both individual patients and public health (3, 4). The rising prevalence of urological disorders and surgical interventions, together with increasing microbial resistance to conventional antibiotics, has further intensified the disease burden of urosepsis (3, 4). Therefore, identifying risk factors for UTI-related bloodstream infections and reliable early markers for high-risk populations is crucial for improving preventive and therapeutic strategies.

The Geriatric Nutritional Risk Index (GNRI) is a validated tool for assessing nutritional status in older adults, requiring only serum albumin levels and body weight for calculation, which makes it simple and efficient (5). Previous studies have demonstrated that GNRI is closely associated with adverse clinical outcomes in patients with infection-related critical illnesses (6–9), highlighting its potential as a practical risk marker for high-risk populations. However, the pathological mechanisms of sepsis vary depending on the infection source. Pulmonary infections often involve alveolar-capillary barrier injury and macrophage overactivation, frequently progressing to acute respiratory distress syndrome. Abdominal infections typically feature bacterial translocation and activation of mixed pathogen-associated molecular patterns, often complicated by intra-abdominal hypertension. In contrast, urinary tract infections are primarily linked to Gram-negative bacterial endotoxins and systemic inflammatory responses triggered by early kidney injury (10–12).

These source-specific pathophysiological features and their associated therapeutic differences highlight the need for individualized, infection-source-based precision medicine in sepsis management. Nevertheless, systematic evidence regarding the predictive value of GNRI specifically in patients with urosepsis remains limited. To address this gap, the present study aims to systematically evaluate the independent prognostic ability of GNRI in this patient population.

## Method and population

### Data sources

This study used two retrospective cohorts. The derivation cohort was obtained from the publicly available Medical Information Mart for Intensive Care IV (MIMIC-IV) database, version 2.2, curated by the Massachusetts Institute of Technology (13). The database includes data from 196,527 adults admitted to Beth Israel Deaconess Medical Center between 2008 and 2019. Access was granted under an approved protocol. Due to the retrospective nature and use of de-identified data, the Massachusetts Institute of Technology Institutional Review Board approved the study and waived the requirement for informed consent.

The external validation cohort consisted of consecutive patients with urosepsis treated at the Department of Urology and Critical Care Medicine, Zhejiang Provincial People’s Hospital Bijie Hospital, China, between January 2015 and January 2025. The hospital’s Ethics Committee approved the study and waived individual informed consent because of the retrospective design.

### Study population

This study focused on patients admitted to the ICU with urosepsis. In both cohorts, sepsis was defined according to the Sepsis-3 consensus criteria, requiring an increase in the Sequential Organ Failure Assessment (SOFA) score by ≥2 points from baseline. The diagnostic criteria for urinary tract infection (UTI) differed between the two cohorts. In the derivation cohort (MIMIC-IV), UTI was identified using ICD-9 and ICD-10 codes. Because ICD coding captures clinical diagnoses of UTI, microbiological confirmation (positive urine culture) was not required. To distinguish urinary-source sepsis from systemic sepsis with secondary urinary findings (e.g., asymptomatic bacteriuria), we additionally required that UTI be the primary diagnosis for ICU admission and that no other predominant infection source (e.g., pneumonia, intra-abdominal infection) be documented as the primary reason for admission, in accordance with Sepsis-3 criteria (14, 15). In the external validation cohort (Zhejiang Provincial People’s Hospital Bijie Hospital), UTI was confirmed by typical clinical presentation (pyuria and/or bacteriuria on urinalysis) and a positive urine culture.

Only patients with an initial admission diagnosis of UTI who also met the Sepsis-3 criteria were enrolled in the urosepsis cohort. All enrolled patients met the following criteria: (1) age ≥ 65 years; (2) survival time and ICU length of stay ≥ 24 h; (3) availability of complete data on body mass index (BMI) and serum albumin levels; and (4) absence of cancer or other conditions affecting serum albumin levels.

### Variable extraction

Data extraction was performed using PostgreSQL (v13.7.2) and Navicat Premium (v16.0) with Structured Query Language (SQL). The extracted variables were grouped into six categories: (1) demographics, (2) comorbidities, (3) vital signs, (4) laboratory parameters, (5) disease severity scores, and (6) treatments. A detailed variable list is available in Supplementary Table S1, S2. For missing data, only variables with <30% missingness were imputed. Multiple imputation was conducted using the R package “mice” (v3.16.0). Notably, the primary exposure variable (GNRI) had no missing data in the final analytic cohort due to the inclusion criteria requiring complete measurements. For other covariates meeting the imputation threshold, five complete datasets were generated through 50 iterations of multiple imputation. The imputation model included all variables used in the primary analysis: continuous variables requiring imputation, fully observed exposure variables (GNRI and its components), the complete outcome variable (28-day mortality), and all other complete categorical and continuous covariates.

### Definition of nutritional status and endpoints

Patients were stratified into four nutritional risk categories based on their GNRI score: no risk (GNRI ≥ 98), low risk (92 ≤ GNRI < 98), moderate risk (82 ≤ GNRI < 92), and high risk (GNRI < 82). The GNRI was calculated using the following formula: GNRI = [1.489 × serum albumin (g/dL)] + [41.7 × (actual body weight / ideal body weight)] (5). Actual body weight was measured at ICU admission. Ideal body weight was defined as the weight corresponding to a body mass index (BMI) of 22 kg/m^2^, calculated as ideal body weight (kg) = 22 × (height in meters)^2^. Of note, the key exposure variable (GNRI) contained no missing data in the final analytic cohort due to inclusion criteria requiring complete measurements.

### Association between GNRI and endpoints

To control for potential confounders, three progressively adjusted models were constructed: Model 1 was unadjusted; Model 2 was adjusted for demographic characteristics (age, gender, race, and BMI) and comorbidities with prognostic significance (those that differed between survivors and non-survivors); Model 3 was further adjusted for clinically relevant variables that differed significantly between survivors and non-survivors, including disease severity scores, key laboratory parameters, and treatment measures. To mitigate multicollinearity that could affect model stability, variance inflation factors (VIFs) were calculated for all variables included in Model 3, and variables with VIF > 5 were excluded. This comprehensive adjustment approach follows a well-established strategy consistently applied in previous studies. The following covariates were ultimately included in Model 3: GNRI, age, gender, BMI, acute kidney injury (AKI), SOFA score, APS III score, INR, total bilirubin (TB), blood urea nitrogen (UREA), non-invasive systolic blood pressure (NBPS), non-invasive diastolic blood pressure (NBPD), platelet count (PLT), red cell distribution width (RDW), red blood cell count (RBC), anion gap (AG), carbon dioxide (CO₂), ionized calcium (Fca), lactate (Lac), pH, partial pressure of oxygen (Po₂), vasopressor use (SA), mechanical ventilation (VP), glucocorticoids (GC), and continuous renal replacement therapy (CRRT).

Subsequently, restricted cubic splines (RCS) with four knots (at the 5th, 35th, 65th, and 95th percentiles) were used to explore potential nonlinear relationships between GNRI and outcomes. Kaplan–Meier (KM) survival curves were employed as a supplementary visualization.

### The incremental effect of GNRI

GNRI was incorporated into six traditional critical illness scoring systems to develop multivariable Cox proportional hazards models. A composite risk score was calculated based on the regression coefficients of each variable using the formula: (*β*₁ × Variable₁) + (β₂ × Variable₂). The predictive ability of the models for adverse outcomes was evaluated using receiver operating characteristic (ROC) curves and the area under the curve (AUC). DeLong’s test was employed to determine whether the improvement in model performance after adding GNRI was statistically significant.

### Statistical analysis

Continuous variables are presented as mean ± standard deviation, and after assessing normality and homogeneity of variance, comparisons were made using Student’s t-test or analysis of variance (ANOVA). Categorical variables are expressed as numbers (percentages) and compared using the Pearson chi-square test or Fisher’s exact test, as appropriate. Covariates for multivariable models were selected based on clinical relevance, prior literature, and significant differences observed in univariate analyses across nutritional risk groups or survival status. Multicollinearity was assessed using VIF, with a threshold of 5. All statistical analyses were performed with R software (version 4.5.1). A two-sided *p*-value < 0.05 was considered statistically significant.

## Results

### Baseline characteristics by nutritional status

A total of 1,218 urosepsis patients were enrolled based on stringent selection criteria. The mean age of the cohort was 77.8 years, and 523 (43.4%) were male. Stratification by GNRI yielded the following results: high-risk (*n* = 544, 44.7%), moderate-risk (*n* = 417, 34.2%), low-risk (*n* = 165, 13.5%), and no-risk (*n* = 92, 7.6%). The baseline characteristics stratified by GNRI category are presented in Table 1. Patients with nutritional risk tended to be older, have a higher burden of comorbidities, and require more intensive treatment. Compared to the no-risk group, high-risk patients exhibited more unstable vital signs, including elevated heart rate, respiratory rate, and systolic blood pressure.

**Table 1:** Summary descriptives table by groups of GNRI group.

Variable	ALL	No	Low	Moderate	High	*p* value
	*N* = 1,218	*N* = 92	*N* = 165	*N* = 417	*N* = 544	
GNRI	84.3 (9.18)	102 (4.38)	94.3 (1.66)	87.1 (2.46)	76.2 (5.10)	<0.001
Age	77.8 (7.83)	76.3 (7.18)	77.5 (8.52)	78.2 (7.50)	77.7 (7.95)	0.140
Gender	523 (42.9%)	44 (47.8%)	69 (41.8%)	189 (45.3%)	221 (40.6%)	0.371
Race	802 (65.8%)	57 (62.0%)	114 (69.1%)	283 (67.9%)	348 (64.0%)	0.392
BMI	28.9 (7.80)	30.2 (6.22)	31.3 (8.53)	29.2 (7.03)	27.8 (8.16)	<0.001
HYT	493 (40.5%)	49 (53.3%)	74 (44.8%)	151 (36.2%)	219 (40.3%)	0.013
AKI	772 (63.4%)	44 (47.8%)	94 (57.0%)	270 (64.7%)	364 (66.9%)	0.001
CKD	404 (33.2%)	22 (23.9%)	55 (33.3%)	161 (38.6%)	166 (30.5%)	0.013
DM	471 (38.7%)	33 (35.9%)	64 (38.8%)	177 (42.4%)	197 (36.2%)	0.241
HF	577 (47.4%)	42 (45.7%)	83 (50.3%)	218 (52.3%)	234 (43.0%)	0.031
MI	154 (12.6%)	8 (8.70%)	28 (17.0%)	53 (12.7%)	65 (11.9%)	0.228
IHD	567 (46.6%)	40 (43.5%)	80 (48.5%)	203 (48.7%)	244 (44.9%)	0.575
COPD	250 (20.5%)	13 (14.1%)	33 (20.0%)	91 (21.8%)	113 (20.8%)	0.426
SOFA	6.82 (3.52)	5.62 (3.29)	6.56 (3.12)	6.67 (3.56)	7.22 (3.57)	<0.001
APSIII	58.1 (21.6)	48.2 (19.9)	52.3 (18.2)	56.4 (20.4)	62.8 (22.6)	<0.001
SAPSII	47.9 (13.3)	42.5 (10.7)	44.9 (12.1)	47.1 (13.4)	50.3 (13.5)	<0.001
OASIS	37.8 (8.36)	35.3 (7.72)	36.1 (9.28)	37.6 (8.41)	39.0 (7.94)	<0.001
CHARLSON	7.04 (2.59)	6.72 (2.54)	6.80 (2.27)	7.20 (2.61)	7.05 (2.66)	0.181
APACHEII	22.6 (7.03)	20.0 (7.05)	21.6 (6.69)	22.4 (6.80)	23.4 (7.18)	<0.001
HR	89.9 (21.7)	81.3 (19.1)	88.4 (22.8)	88.9 (21.4)	92.6 (21.5)	<0.001
NBPS	120 (26.7)	130 (26.0)	125 (25.5)	120 (27.0)	118 (26.6)	<0.001
NBPD	67.2 (20.6)	68.5 (16.1)	69.6 (20.6)	66.8 (19.1)	66.6 (22.3)	0.360
RR	19.9 (6.63)	18.3 (5.65)	20.2 (7.97)	19.6 (6.16)	20.3 (6.64)	0.017
SpO2	96.4 (4.89)	97.1 (3.98)	95.7 (4.76)	96.4 (5.33)	96.5 (4.69)	0.095
HCT	32.0 (6.48)	33.5 (6.94)	33.4 (6.49)	32.5 (6.56)	30.8 (6.16)	<0.001
Hb	10.3 (2.16)	11.0 (2.41)	10.8 (2.17)	10.5 (2.17)	9.92 (2.03)	<0.001
PLT	211 (115)	199 (102)	202 (98.9)	203 (104)	222 (128)	0.045
RDW	15.8 (2.43)	15.0 (2.55)	15.6 (2.39)	15.6 (2.23)	16.2 (2.51)	<0.001
RBC	3.47 (0.74)	3.64 (0.83)	3.65 (0.75)	3.53 (0.74)	3.34 (0.71)	<0.001
WBC	12.0 [8.60;16.7]	10.6 [8.33;13.5]	11.1 [8.10;14.3]	12.0 [8.50;16.6]	13.2 [8.97;17.9]	<0.001
ALB	2.91 (0.59)	4.04 (0.30)	3.55 (0.14)	3.07 (0.18)	2.39 (0.34)	<0.001
AG	15.5 (4.61)	15.8 (4.61)	15.3 (4.17)	15.5 (4.53)	15.5 (4.80)	0.835
Ca	8.30 (1.00)	8.87 (0.97)	8.73 (0.78)	8.39 (1.05)	8.01 (0.91)	<0.001
Cl	104 (7.45)	102 (6.65)	103 (6.80)	104 (7.53)	105 (7.65)	0.006
Glu	159 (83.1)	156 (81.6)	157 (63.0)	165 (92.6)	157 (81.0)	0.496
K	4.25 (0.80)	4.23 (0.75)	4.26 (0.85)	4.29 (0.77)	4.22 (0.81)	0.626
CO2	24.0 (6.40)	25.5 (6.51)	25.0 (6.14)	24.2 (6.32)	23.2 (6.42)	<0.001
Lac	2.40 (1.99)	2.23 (1.55)	2.14 (1.53)	2.36 (1.97)	2.54 (2.18)	0.054
PCo2	42.0 (12.6)	42.5 (12.6)	43.3 (14.0)	42.7 (13.5)	41.0 (11.5)	0.076
pH	7.35 (0.11)	7.38 (0.09)	7.36 (0.10)	7.35 (0.10)	7.34 (0.12)	0.020
Po2	130 (106)	141 (105)	140 (127)	133 (109)	122 (95.4)	0.135
INR	1.30 [1.20;1.70]	1.30 [1.10;1.72]	1.30 [1.20;1.70]	1.30 [1.20;1.70]	1.40 [1.20;1.70]	0.454
PT	14.7 [13.0;18.2]	14.4 [12.5;18.9]	14.3 [13.0;18.1]	14.6 [12.9;18.1]	14.9 [13.2;18.1]	0.503
APTT	32.2 [27.7;41.2]	31.2 [26.3;36.1]	30.9 [26.9;40.4]	31.9 [27.8;41.7]	33.0 [28.1;41.2]	0.077
ALT	25.0 [14.0;50.0]	22.5 [16.0;41.2]	22.0 [14.0;43.0]	25.0 [14.0;50.0]	26.0 [15.0;52.2]	0.480
AST	39.0 [23.0;82.0]	39.0 [22.8;77.2]	36.0 [24.0;72.0]	39.0 [22.0;78.0]	41.0 [23.0;85.0]	0.727
TB	0.60 [0.40;1.10]	0.70 [0.48;1.22]	0.70 [0.40;1.10]	0.60 [0.40;1.10]	0.60 [0.30;1.10]	0.130
CRE	1.30 [0.90;2.10]	1.10 [0.70;1.60]	1.20 [0.80;1.90]	1.30 [0.90;2.20]	1.30 [0.80;2.10]	0.009
URE	28.0 [18.0;48.0]	20.0 [14.0;28.2]	25.0 [16.0;46.0]	27.0 [18.0;49.0]	32.0 [19.0;51.0]	<0.001
LDH	294 [217;428]	297 [218;414]	301 [223;424]	286 [214;425]	292 [218;432]	0.910
SA	971 (79.7%)	71 (77.2%)	129 (78.2%)	328 (78.7%)	443 (81.4%)	0.596
VP	877 (72.0%)	59 (64.1%)	105 (63.6%)	289 (69.3%)	424 (77.9%)	<0.001
GC	398 (32.7%)	27 (29.3%)	49 (29.7%)	127 (30.5%)	195 (35.8%)	0.208
Ventilation	1,132 (92.9%)	88 (95.7%)	157 (95.2%)	393 (94.2%)	494 (90.8%)	0.069
CRRT	143 (11.7%)	10 (10.9%)	17 (10.3%)	50 (12.0%)	66 (12.1%)	0.918
Hosp dead	209 (17.2%)	5 (5.43%)	12 (7.27%)	58 (13.9%)	134 (24.6%)	<0.001
Hosp time	19.6 (19.3)	21.1 (20.4)	17.6 (15.5)	19.7 (19.9)	19.9 (19.6)	0.382
ICU dead	228 (18.7%)	7 (7.61%)	13 (7.88%)	60 (14.4%)	148 (27.2%)	<0.001
ICU time	8.68 (9.43)	9.68 (10.8)	8.53 (8.47)	8.54 (9.15)	8.66 (9.69)	0.817

AKI, acute kidney injury; ALB, albumin; ALT, alanine aminotransferase; APACHE II, Acute Physiology and Chronic Health Evaluation II; APS III, Acute Physiology Score III; AST, aspartate aminotransferase; BMI, body mass index; Ca, calcium; CHARLSON, Charlson Comorbidity Index; CKD, chronic kidney disease; Cl, chloride; COPD, chronic obstructive pulmonary disease; CRRT, continuous renal replacement therapy; DM, diabetes mellitus; GC, glucocorticoids; GNRI, Geriatric Nutritional Risk Index; Hb, hemoglobin; HCT, hematocrit; HF, heart failure; HR, heart rate; HYT, hypertension; IHD, ischemic heart disease; INR, international normalized ratio; K, potassium; LDH, lactate dehydrogenase; MI, myocardial infarction; NBPD, non-invasive blood pressure diastolic; NBPS, non-invasive blood pressure systolic; OASIS, Oxford Acute Severity of Illness Score; PLT, platelet; PT, prothrombin time; RBC, red blood cell; RDW, red cell distribution width; RR, respiratory rate; SA, vasopressors; SAPS II, Simplified Acute Physiology Score II; SOFA, Sequential Organ Failure Assessment; SpO₂, oxygen saturation; TB, total bilirubin; URE, urea; VP, ventilation; WBC, white blood cell.

Significant differences were also observed in numerous laboratory parameters. The high-risk group had increased levels of platelets, red cell distribution width (RDW), white blood cells (WBC), chloride, creatinine, and blood urea nitrogen. Conversely, decreased levels were found for partial pressure of oxygen (PO₂), hematocrit (HCT), hemoglobin, red blood cell count, albumin, anion gap, bicarbonate, serum calcium, and PH. Furthermore, clinical severity scores were positively correlated with the degree of nutritional risk. Regarding clinical outcomes, the high-risk group had significantly higher mortality rates compared to the no-risk group for both 28-day ICU mortality (27.2% vs. 7.6%, *p* < 0.001) and in-hospital mortality (24.6% vs. 5.43%, *p* < 0.001).

### Association between GNRI and short-term mortality

As shown in Table 2, all three multivariable Cox proportional hazards models demonstrated a significant inverse association between higher GNRI scores and 28-day ICU mortality: Model 1 (unadjusted: HR = 0.95, 95% CI: 0.93–0.96, *p* < 0.001), Model 2 (partial adjustment: HR = 0.95, 95% CI: 0.94–0.96, *p* < 0.001), and Model 3 (fully adjusted: HR = 0.96, 95% CI: 0.94–0.98, *p* < 0.001). Similarly, patients at high nutritional risk had significantly increased mortality across all three models compared to the no-risk group: Model 1 (HR = 3.87, 95% CI: 1.81–8.26, *p* < 0.001), Model 2 (HR = 3.02, 95% CI: 1.40–6.51, *p* = 0.005), and Model 3 (HR = 2.34, 95% CI: 1.07–5.11, *p* = 0.034).

**Table 2:** The relationship between GNRI and short-term mortality rate in ICU (internal discovery queue).

Characteristic	Model 1			Model 2			Model 3		
	HR	95% CI	*p*-value	HR	95% CI	*p*-value	HR	95% CI	*p*-value
GNRI	0.95	0.93, 0.96	<0.001	0.95	0.94, 0.96	<0.001	0.96	0.94, 0.98	<0.001
GNRI group									
No	Ref	Ref		Ref	Ref		Ref	Ref	
Low	1.11	0.44, 2.79	0.8	0.98	0.39, 2.47	>0.9	1.00	0.40, 2.54	>0.9
Moderate	2.05	0.94, 4.49	0.072	1.61	0.73, 3.54	0.2	1.49	0.67, 3.30	0.3
High	3.87	1.81, 8.26	<0.001	3.02	1.40, 6.51	0.005	2.34	1.07, 5.11	0.034
*p* for trend		<0.001			<0.001			<0.001	

CI, confidence interval; GNRI, Geriatric Nutritional Risk Index; HR, hazard ratio; ICU, intensive care unit. Model 1: unadjusted; Model 2: adjusted for age, gender, race, BMI, and comorbidities; Model 3: fully adjusted for demographics, comorbidities, severity scores, laboratory parameters, and treatments. *p* for trend calculated across GNRI risk categories.

**Figure 1:**
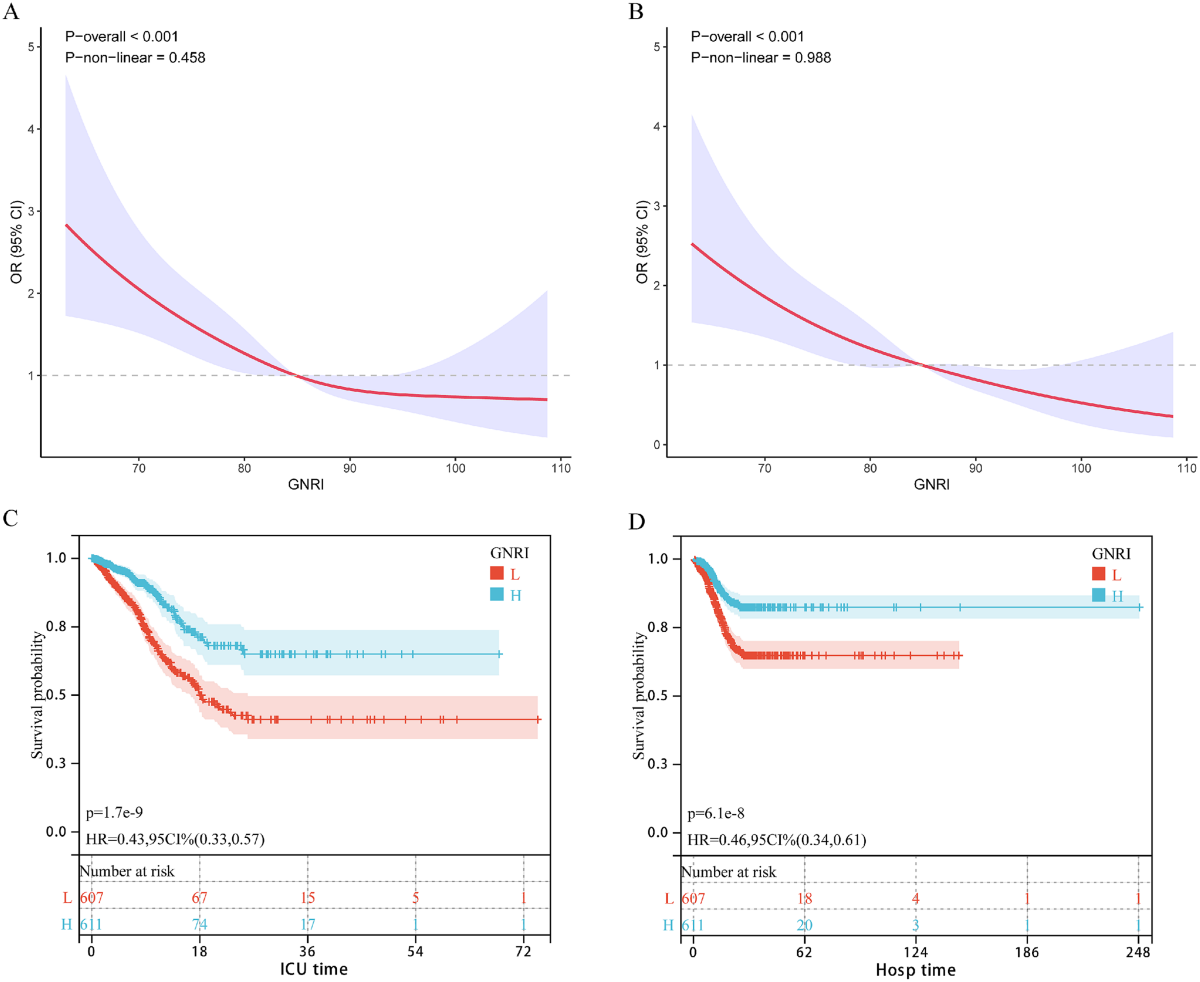
Correlation between GNRI and short-term mortality in patients with urosepsis (internal discovery cohort). Restricted cubic spline curves show a significant nonlinear inverse dose–response relationship between GNRI and ICU **(A)** and in-hospital **(B)** mortality. Kaplan–Meier survival curves demonstrate significantly lower survival rates in patients with higher GNRI for both ICU **(C)** and in-hospital **(D)** mortality.

Consistent results were observed for 28-day in-hospital all-cause mortality (Table 3). Higher GNRI scores were consistently associated with reduced mortality risk in all three models: Model 1 (HR = 0.95, 95% CI: 0.93–0.96, *p* < 0.001), Model 2 (HR = 0.95, 95% CI: 0.94–0.96, *p* < 0.001), and Model 3 (HR = 0.96, 95% CI: 0.94–0.98, *p* < 0.001). Likewise, the high-risk group consistently exhibited elevated mortality compared to the no-risk group: Model 1 (HR = 4.79, 95% CI: 1.96–11.7, *p* < 0.001), Model 2 (HR = 3.79, 95% CI: 1.54–9.32, *p* = 0.004), and Model 3 (HR = 2.63, 95% CI: 1.05–6.58, *p* = 0.039). RCS analysis revealed a linear inverse dose–response relationship between GNRI and short-term mortality in urosepsis patients (Figures 1A,B). Kaplan–Meier survival analysis confirmed that higher GNRI scores were associated with lower short-term mortality (Figures 1C,D).

**Table 3:** The relationship between GNRI and short-term mortality rate in hospital (internal discovery queue).

Characteristic	Model 1			Model 2			Model 3		
	HR	95% CI	*p*-value	HR	95% CI	*p*-value	HR	95% CI	*p*-value
GNRI	0.95	0.93, 0.96	<0.001	0.95	0.94, 0.96	<0.001	0.96	0.94, 0.98	<0.001
GNRI group									
No	Ref	Ref		Ref	Ref		Ref	Ref	
Low	1.46	0.52, 4.15	0.5	1.27	0.45, 3.63	0.6	1.20	0.42, 3.45	0.7
Moderate	2.64	1.06, 6.58	0.037	2.15	0.86, 5.38	0.10	1.74	0.69, 4.40	0.2
High	4.79	1.96, 11.7	<0.001	3.79	1.54, 9.32	0.004	2.63	1.05, 6.58	0.039
*p* for trend		<0.001			<0.001			<0.001	

CI, confidence interval; GNRI, Geriatric Nutritional Risk Index; HR, hazard ratio; ICU, intensive care unit. Model 1: unadjusted; Model 2: adjusted for age, gender, race, BMI, and comorbidities; Model 3: fully adjusted for demographics, comorbidities, severity scores, laboratory parameters, and treatments. *p* for trend calculated across GNRI risk categories.

### Incremental effect of GNRI

**Figure 2:**
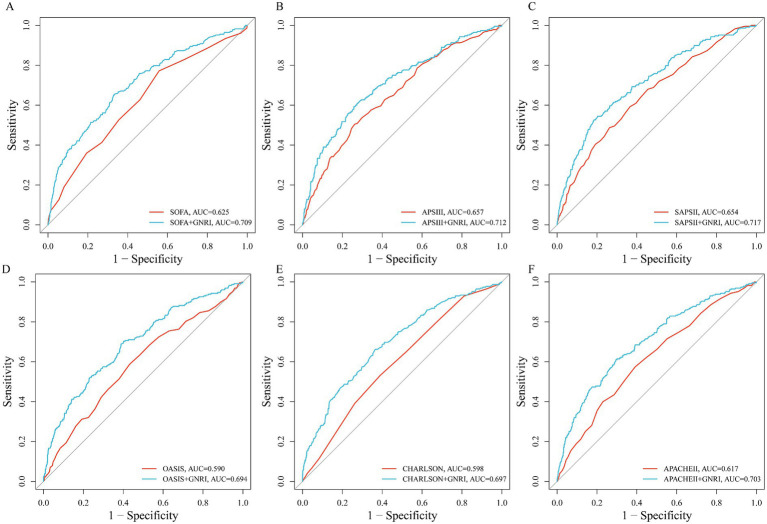
The incremental effect of GNRI; Adding GNRI to six established severity scores (SOFA, APS III, SAPS II, OASIS, Charlson, APACHE II) consistently improved their discriminatory performance, as shown by increased area under the receiver operating characteristic curves **(A–F)**.

To evaluate the added value of the GNRI, we tested its combination with six established severity scores (SOFA, APS III, SAPS II, OASIS, Charlson, APACHE II) for predicting 28-day ICU mortality. The inclusion of GNRI universally enhanced the predictive accuracy of all models (Figures 2A–F). DeLong’s test was used to compare the area under the receiver operating characteristic curves (AUCs) of models with and without GNRI, confirming that the improvements were statistically significant for all scores (SOFA: AUC increased from 0.625 to 0.709, *p* < 0.001; APS III: 0.657 to 0.717, *p* = 0.001; SAPS II: 0.654 to 0.717, *p* = 0.001; OASIS: 0.590 to 0.694, *p* < 0.001; Charlson: 0.598 to 0.697, *p* < 0.001; APACHE II: 0.617 to 0.703, *p* < 0.001). These findings indicate that the addition of GNRI significantly improved model discrimination beyond that achieved by established severity scores alone.

### Subgroup analysis

We conducted subgroup analyses and interaction tests to examine whether demographic characteristics and comorbidities modify the association between GNRI and 28-day ICU/in-hospital mortality. After multivariable adjustment, the results (Supplementary Figures S1, S2) showed that the association between GNRI and mortality remained consistent across the vast majority of subgroups. Although statistical significance was not reached in some subgroups (e.g., patients with BMI ≥ 30, acute kidney injury, or diabetes), the trend and direction of the hazard ratios (HRs) in these subgroups were aligned with the main analysis. This is likely attributable to the relatively small sample sizes and lower event rates in these specific subgroups, which may have resulted in inadequate statistical power. Therefore, the non-significant associations observed in these subgroup analyses should be interpreted with caution, as they may reflect the risk of type II error rather than a true absence of effect. Furthermore, interaction tests did not reveal any significant interaction between GNRI and demographic or clinical characteristics, supporting that the lack of statistical significance in other subgroups is likely due to sample size limitations.

### External cohort validation

**Figure 3:**
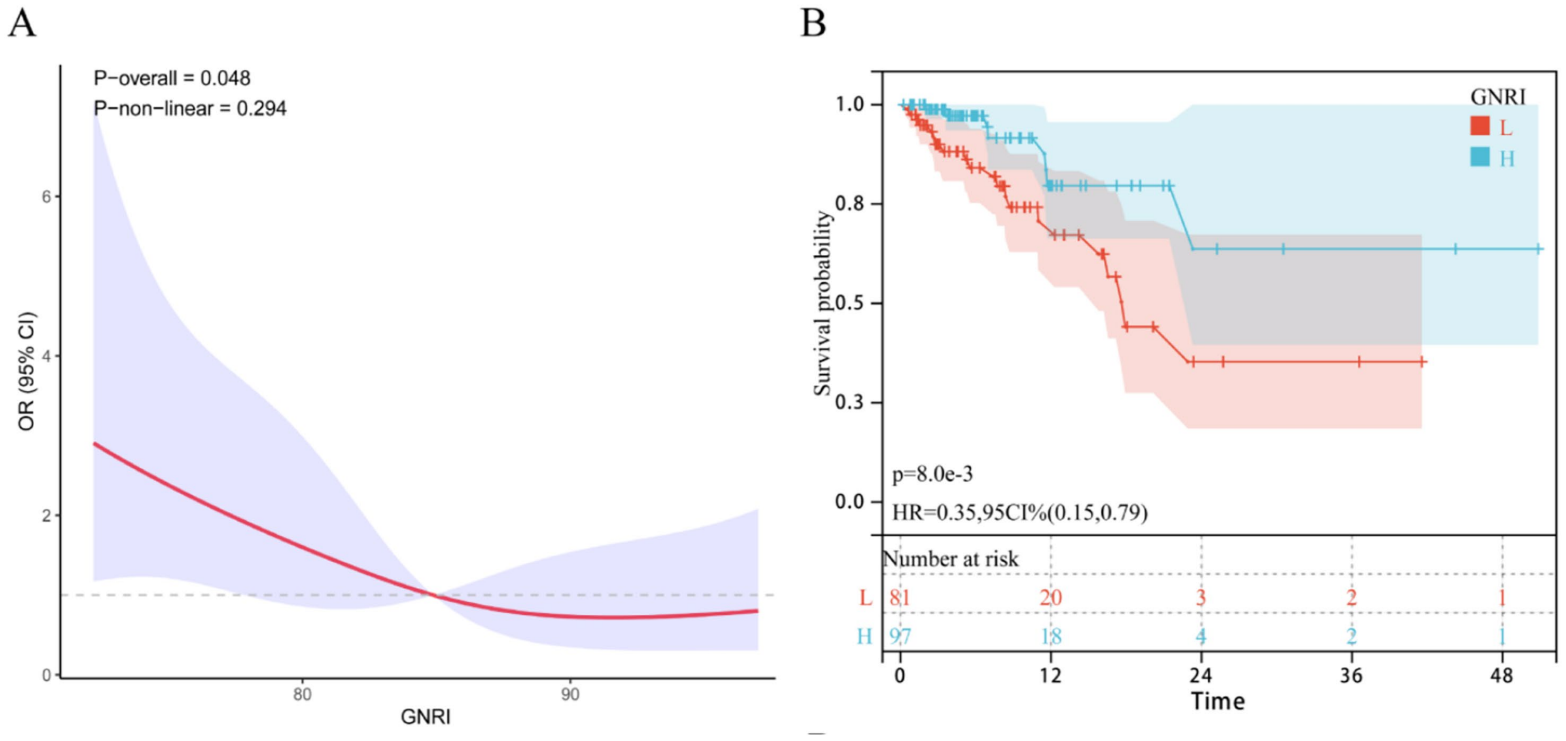
The correlation between GNRI and short-term mortality in patients with urinary sepsis (External reality queue); Kaplan–Meier analysis **(A)** confirms significantly higher mortality in patients with GNRI above the median. The restricted cubic spline curve **(B)** reveals a significant nonlinear inverse dose–response relationship between GNRI and ICU mortality.

The external validation cohort comprised 178 patients with urosepsis (28-day mortality rate = 16.3%). In a Cox regression model adjusted for all potential covariates, a higher GNRI score was significantly associated with a reduced risk of 28-day mortality (HR = 0.95; 95% CI: 0.91–1.00, *p* = 0.033). Subsequently, Kaplan–Meier analysis indicated that patients with a GNRI score above the cohort median had a significantly lower risk of mortality (log-rank *p* = 0.008; HR = 0.35, 95% CI: 0.15–0.79; Figure 3A). Furthermore, the RCS curve confirmed a significant inverse dose–response relationship between GNRI and mortality (*p* = 0.048; Figure 3B).

## Discussion

Urosepsis is a severe complication of urinary tract infection, and its incidence has been steadily increasing in recent years. This trend is closely linked to the rising prevalence of urological diseases and surgical interventions, along with growing microbial resistance to conventional antibiotics, posing substantial challenges to both individual patients and public health systems (1–4). Previous studies have emphasized the need to identify reliable and easily applicable biomarkers to improve outcomes in this population (1–7, 16–18).

The GNRI is a simple and effective nutritional assessment tool widely used to screen for malnutrition risk and predict clinical prognosis in older patients (5). Numerous studies have confirmed its predictive value in ICU settings, demonstrating that low GNRI scores are strongly associated with increased mortality and poor outcomes across various critical conditions, including sepsis, trauma, and acute kidney injury (6–9). However, the pathological mechanisms of sepsis vary considerably depending on the infection source. Pulmonary infections often involve alveolar-capillary barrier injury and macrophage overactivation, frequently progressing to acute respiratory distress syndrome. Abdominal infections typically feature bacterial translocation and activation of mixed pathogen-associated molecular patterns, often complicated by intra-abdominal hypertension. In contrast, urinary tract infections are primarily associated with systemic inflammatory responses induced by Gram-negative bacterial endotoxins and early kidney injury (10–12). These source-specific pathophysiological features and their corresponding therapeutic differences highlight the need for individualized, infection-source-based precision medicine in sepsis management.

To this end, this study is the first to demonstrate an association between GNRI and adverse outcomes in older patients with urosepsis. Our results show that lower GNRI levels independently predict 28-day all-cause mortality both in the ICU and during hospitalization. Moreover, we observed a linear inverse correlation between GNRI and early mortality in this population, and this relationship remained consistent across the vast majority of demographic and comorbidity subgroups. All key findings were further validated in an external real-world cohort. Notably, adding GNRI improved the AUC of the SOFA score by 0.084 (from 0.625 to 0.709), representing a moderate but clinically meaningful enhancement in discriminative performance. This improvement suggests that incorporating nutritional assessment may facilitate more accurate risk stratification in older patients with urosepsis. This evidence highlights the critical importance of early nutritional intervention in this population.

Malnutrition is defined as a state in which energy intake fails to meet the body’s physiological demands, and it is closely associated with the onset and progression of various severe diseases, including sepsis (19–21). The underlying mechanisms are multifactorial, with the inflammatory response considered a core mediator (22, 23). Extensive experimental research indicates that the core pathological process of sepsis initiation and progression unfolds in three phases: excessive activation of the immune-inflammatory response, followed by a compensatory anti-inflammatory response, and subsequent immunosuppression (24–26). A significant bidirectional interaction exists between malnutrition and inflammation. Hypoalbuminemia, a marker of malnutrition, is associated with systemic hyperinflammation (26, 27). Conversely, sustained inflammation suppresses albumin synthesis, further exacerbating malnutrition and thereby creating a vicious cycle (28, 29). In addition, malnutrition can trigger oxidative stress, lipid peroxidation, immune dysregulation, and programmed cell death (30, 31). Collectively, these mechanisms aggravate tissue and organ damage in sepsis (32).

In addition to the mechanisms shared with sepsis in general, malnutrition may specifically influence the occurrence and progression of urosepsis through several distinct pathways. First, malnutrition directly impairs the local defense barrier of the urinary tract. The synthesis of the protective glycoprotein layer on the urothelial surface depends on adequate nutrition; deficiency can lead to thinning or disruption of this layer, thereby enhancing bacterial adhesion to epithelial cells (33, 34). Second, deficiencies in specific nutrients may increase the virulence of pathogens such as uropathogenic *Escherichia coli*, thereby exacerbating the infection (35). Third, normal urinary pH is a key determinant of bacterial invasiveness. Malnutrition, often accompanied by metabolic disturbances, can result in abnormal urinary acidity, altering the local microenvironment and promoting bacterial colonization (36, 37). Finally, malnourished patients have a higher incidence of urinary stones. Stones can serve as a nidus for pathogen retention and cause urinary tract obstruction, a major risk factor for the development and deterioration of urosepsis (38).

Although this study clarifies the independent prognostic value of GNRI for short-term outcomes in patients with urosepsis, several limitations should be acknowledged. First, the retrospective design precludes causal inference, and a causal relationship between GNRI and mortality cannot be established. Second, despite careful adjustment for a wide range of clinically relevant covariates, the possibility of residual or unmeasured confounding cannot be entirely excluded, as with all observational studies. Third, the accuracy of variables derived from the databases (e.g., dietary adequacy, nutritional biomarkers) is subject to inherent limitations of secondary data sources, and we were unable to verify the completeness or precision of these records. Fourth, GNRI was assessed only once at ICU admission. However, the nutritional status of critically ill patients is dynamic, and a single time-point measurement may not fully capture subsequent nutritional deterioration or recovery. Specifically: ① Fluid balance may affect both albumin and body weight measurements. Patients with urosepsis often receive fluid resuscitation, and albumin dilution or weight gain due to fluid overload may falsely lower GNRI independently of true nutritional status. ② This study did not collect data on nutritional interventions during hospitalization (e.g., enteral or parenteral nutrition). Such interventions could modify both GNRI components (albumin, body weight) and clinical outcomes, representing unmeasured confounders. ③ Because GNRI was calculated only at baseline, we cannot determine whether changes in nutritional status over time (e.g., improvement after nutritional support or deterioration due to persistent inflammation) mediate or modify the prognostic effect. Therefore, future studies should employ repeated GNRI measurements, combined with fluid-corrected albumin or dry body weight. Fifth, constrained by database information, this study could not incorporate some key clinical details, such as the specific type of urological surgery, causative pathogen species, and whether mixed infections occurred during hospitalization. Sixth, although we used DeLong’s test to compare AUCs and demonstrated that adding GNRI significantly improved the discriminative ability of established severity scores, we did not calculate the net reclassification improvement (NRI) or the integrated discrimination improvement (IDI), both of which are currently recommended as standard metrics for evaluating incremental prognostic value. Future studies should incorporate NRI and IDI to further validate the reclassification performance of GNRI. Seventh, the external validation cohort was relatively small (*n* = 178) and single-center in design, which may affect model robustness and calibration reliability. Larger, multicenter prospective validation cohorts are needed to confirm the prognostic value of GNRI and to assess calibration performance. Finally, future prospective, multicenter studies incorporating serial nutritional assessments and detailed clinical variables are warranted to further validate our findings.

## Conclusion

This study demonstrates that GNRI is independently and inversely associated with short-term all-cause mortality in older patients with urosepsis. Lower GNRI levels significantly predict increased 28-day ICU and in-hospital mortality, exhibiting a clear dose–response relationship. Moreover, adding GNRI to established critical illness severity scores improves their prognostic accuracy, supporting its utility as a simple and clinically feasible risk-stratification tool. These findings highlight the importance of early nutritional assessment and intervention to improve outcomes in high-risk patients with urosepsis. Future prospective, multicenter studies are warranted to validate these results and further establish the role of GNRI in guiding individualized management.

## Data Availability

The original contributions presented in the study are included in the article/supplementary material, further inquiries can be directed to the corresponding authors.

## Supplementary material

SUPPLEMENTARY FIGURE S1 Subgroup and interaction analysis of the relationship between GNRI and short-term mortality in patients with urinary sepsis in ICU.

SUPPLEMENTARY FIGURE S2 Subgroup and interaction analysis of the relationship between GNRI and short-term mortality in patients with urinary sepsis in Hosp.
