# Supplementary figures and images for: The geriatric nutritional risk index predicts short-term mortality in older patients with urosepsis: a retrospective cohort study with external validation
Source: Front Nutr. 2026 Jul 2;13:1793046. doi: 10.3389/fnut.2026.1793046 (PMC13374418; doi:10.3389/fnut.2026.1793046)

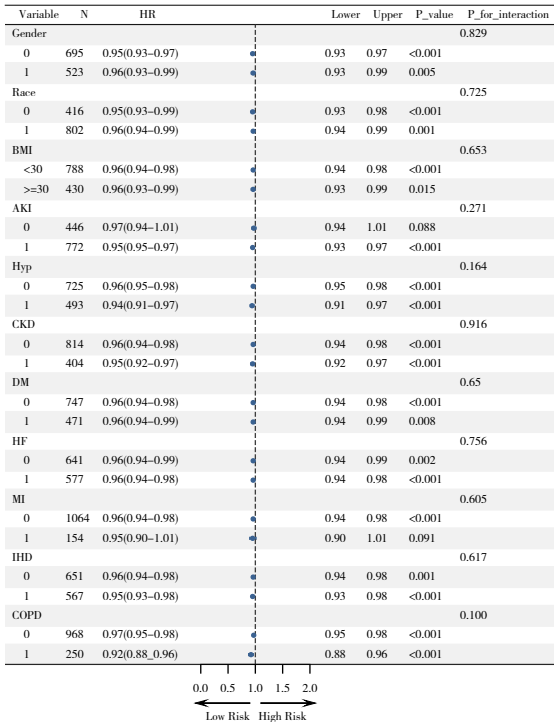

Supplement: SUPPLEMENTARY FIGURE S1 — Subgroup and interaction analysis of the relationship between GNRI and short-term mortality in patients with urinary sepsis in ICU. [file Image_1.pdf]

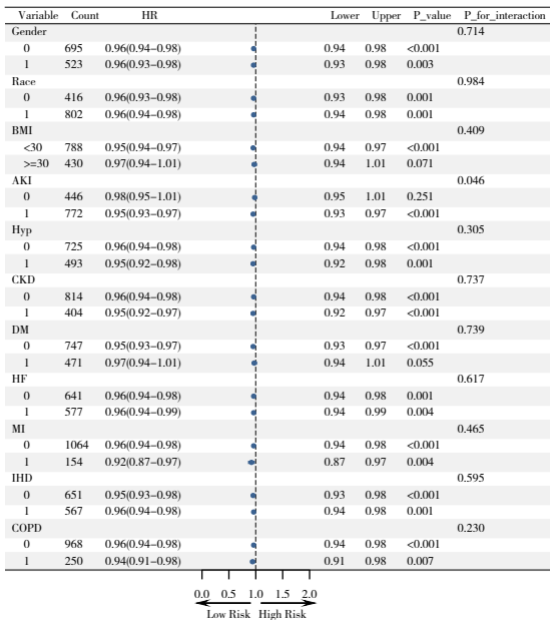

Supplement: SUPPLEMENTARY FIGURE S2 — Subgroup and interaction analysis of the relationship between GNRI and short-term mortality in patients with urinary sepsis in Hosp. [file Image_2.pdf]
