# Supplementary material for: The geriatric nutritional risk index predicts short-term mortality in older patients with urosepsis: a retrospective cohort study with external validation
Source: Front Nutr. 2026 Jul 2;13:1793046. doi: 10.3389/fnut.2026.1793046 (PMC13374418; doi:10.3389/fnut.2026.1793046)
Supplement: Supplementary file 3 [file Table_1.docx]

Summary descriptives table by groups of ICU dead

|  | **ALL** | **Survivor** | **No-survivor** | **P value** |
| --- | --- | --- | --- | --- |
|  | ***N=1932*** | ***N=1631*** | ***N=301*** |  |
| Age | 69.1 (15.1) | 68.5 (15.1) | 71.8 (14.5) | <0.001 |
| Gender: | 839 (43.4%) | 724 (44.4%) | 115 (38.2%) | 0.054 |
| Race: | 1219 (63.1%) | 1043 (63.9%) | 176 (58.5%) | 0.081 |
| BMI | 29.9 (8.96) | 29.9 (8.96) | 29.4 (8.96) | 0.366 |
| Hyp: | 724 (37.5%) | 626 (38.4%) | 98 (32.6%) | 0.064 |
| AKI: | 1207 (62.5%) | 977 (59.9%) | 230 (76.4%) | <0.001 |
| CKD: | 521 (27.0%) | 428 (26.2%) | 93 (30.9%) | 0.109 |
| DM: | 710 (36.7%) | 608 (37.3%) | 102 (33.9%) | 0.291 |
| HF: | 742 (38.4%) | 617 (37.8%) | 125 (41.5%) | 0.251 |
| MI: | 202 (10.5%) | 164 (10.1%) | 38 (12.6%) | 0.216 |
| IHD: | 726 (37.6%) | 614 (37.6%) | 112 (37.2%) | 0.937 |
| COPD: | 337 (17.4%) | 277 (17.0%) | 60 (19.9%) | 0.247 |
| SOFA | 7.07 (3.77) | 6.78 (3.60) | 8.67 (4.26) | <0.001 |
| APSIII | 58.9 (22.4) | 56.9 (21.5) | 70.0 (23.8) | <0.001 |
| SIRS | 2.84 (0.91) | 2.83 (0.91) | 2.92 (0.87) | 0.085 |
| SAPSII | 45.1 (14.1) | 43.8 (13.5) | 52.1 (14.8) | <0.001 |
| OASIS | 36.8 (8.46) | 36.3 (8.31) | 39.2 (8.84) | <0.001 |
| Charlson | 6.06 (2.87) | 5.91 (2.86) | 6.90 (2.76) | <0.001 |
| APACHEII | 21.9 (7.36) | 21.4 (7.23) | 24.6 (7.43) | <0.001 |
| HR | 91.9 (21.8) | 91.8 (22.1) | 92.5 (20.2) | 0.607 |
| NBPS | 120 (26.1) | 121 (26.0) | 116 (26.0) | <0.001 |
| NBPD | 68.4 (20.2) | 68.8 (20.1) | 66.2 (20.6) | 0.046 |
| RR | 20.1 (6.52) | 20.0 (6.59) | 20.5 (6.14) | 0.218 |
| Spo2 | 96.5 (4.74) | 96.6 (4.69) | 96.1 (4.98) | 0.095 |
| HCT | 31.7 (6.72) | 31.8 (6.64) | 31.0 (7.10) | 0.056 |
| Hb | 10.3 (2.25) | 10.3 (2.22) | 10.1 (2.35) | 0.085 |
| PLT | 204 (116) | 206 (116) | 190 (120) | 0.029 |
| RDW | 16.0 (2.68) | 15.8 (2.55) | 16.8 (3.17) | <0.001 |
| RBC | 3.45 (0.80) | 3.47 (0.78) | 3.34 (0.86) | 0.013 |
| WBC | 13.9 (11.7) | 13.6 (11.0) | 15.3 (14.6) | 0.064 |
| ALB | 2.90 (0.62) | 2.93 (0.61) | 2.73 (0.66) | <0.001 |
| AG | 15.6 (4.91) | 15.5 (4.89) | 16.3 (4.98) | 0.012 |
| TCa | 8.26 (1.03) | 8.27 (1.04) | 8.23 (0.99) | 0.513 |
| Cl | 104 (7.84) | 104 (7.69) | 103 (8.63) | 0.182 |
| Glu | 158 (86.4) | 158 (85.8) | 154 (89.9) | 0.440 |
| K | 4.23 (0.82) | 4.22 (0.81) | 4.30 (0.84) | 0.126 |
| CO2 | 23.9 (6.40) | 24.0 (6.46) | 23.2 (6.03) | 0.039 |
| FCa | 1.11 (0.13) | 1.11 (0.13) | 1.09 (0.12) | 0.037 |
| Lac | 2.45 (2.14) | 2.41 (2.14) | 2.67 (2.10) | 0.048 |
| PCo2 | 42.0 (12.6) | 42.0 (12.5) | 42.1 (12.9) | 0.853 |
| PH | 7.35 (0.11) | 7.35 (0.11) | 7.34 (0.11) | 0.020 |
| Po2 | 127 (103) | 130 (105) | 114 (88.7) | 0.005 |
| INR | 1.67 (1.04) | 1.64 (1.02) | 1.84 (1.14) | 0.005 |
| PT | 18.1 (10.9) | 17.8 (10.6) | 19.9 (12.0) | 0.004 |
| APTT | 40.6 (25.4) | 40.2 (25.6) | 42.8 (24.4) | 0.086 |
| ALT | 152 (680) | 157 (714) | 122 (451) | 0.273 |
| AST | 277 (1310) | 282 (1351) | 248 (1066) | 0.620 |
| TB | 2.15 (5.00) | 1.85 (4.13) | 3.77 (8.06) | <0.001 |
| CRE | 1.81 (1.75) | 1.78 (1.75) | 1.99 (1.75) | 0.059 |
| UREA | 36.1 (29.0) | 34.7 (28.0) | 44.0 (32.5) | <0.001 |
| LDH | 599 (1392) | 570 (1321) | 755 (1721) | 0.078 |
| SA: | 1571 (81.3%) | 1308 (80.2%) | 263 (87.4%) | 0.004 |
| VP: | 1376 (71.2%) | 1129 (69.2%) | 247 (82.1%) | <0.001 |
| GC: | 672 (34.8%) | 549 (33.7%) | 123 (40.9%) | 0.019 |
| Ventilation: | 1792 (92.8%) | 1517 (93.0%) | 275 (91.4%) | 0.372 |
| CRRT: | 281 (14.5%) | 206 (12.6%) | 75 (24.9%) | <0.001 |
| GNRI | 84.2 (9.54) | 84.7 (9.29) | 81.4 (10.3) | <0.001 |
| GNRI group: |  |  |  | <0.001 |
| No | 151 (7.82%) | 135 (8.28%) | 16 (5.32%) |  |
| Low | 260 (13.5%) | 228 (14.0%) | 32 (10.6%) |  |
| Moderate | 682 (35.3%) | 605 (37.1%) | 77 (25.6%) |  |
| High | 839 (43.4%) | 663 (40.6%) | 176 (58.5%) |  |
