# Supplementary material for: The geriatric nutritional risk index predicts short-term mortality in older patients with urosepsis: a retrospective cohort study with external validation
Source: Front Nutr. 2026 Jul 2;13:1793046. doi: 10.3389/fnut.2026.1793046 (PMC13374418; doi:10.3389/fnut.2026.1793046)
Supplement: Supplementary file 4 [file Table_2.docx]

Summary descriptives table by groups of Hosp dead

|  | **ALL** | **Survivor** | **No-survivor** | **P value** |
| --- | --- | --- | --- | --- |
|  | ***N=1932*** | ***N=1657*** | ***N=275*** |  |
| Age | 69.1 (15.1) | 68.5 (15.1) | 72.5 (14.3) | <0.001 |
| Gender: | 839 (43.4%) | 734 (44.3%) | 105 (38.2%) | 0.067 |
| Race: | 1219 (63.1%) | 1061 (64.0%) | 158 (57.5%) | 0.043 |
| BMI | 29.9 (8.96) | 30.0 (8.95) | 29.2 (9.01) | 0.225 |
| Hyp: | 724 (37.5%) | 632 (38.1%) | 92 (33.5%) | 0.156 |
| AKI: | 1207 (62.5%) | 996 (60.1%) | 211 (76.7%) | <0.001 |
| CKD: | 521 (27.0%) | 434 (26.2%) | 87 (31.6%) | 0.070 |
| DM: | 710 (36.7%) | 622 (37.5%) | 88 (32.0%) | 0.090 |
| HF: | 742 (38.4%) | 625 (37.7%) | 117 (42.5%) | 0.145 |
| MI: | 202 (10.5%) | 164 (9.90%) | 38 (13.8%) | 0.063 |
| IHD: | 726 (37.6%) | 621 (37.5%) | 105 (38.2%) | 0.876 |
| COPD: | 337 (17.4%) | 281 (17.0%) | 56 (20.4%) | 0.196 |
| SOFA | 7.07 (3.77) | 6.81 (3.62) | 8.64 (4.22) | <0.001 |
| APSIII | 58.9 (22.4) | 57.1 (21.6) | 69.8 (24.0) | <0.001 |
| SIRS | 2.84 (0.91) | 2.82 (0.91) | 2.96 (0.85) | 0.016 |
| SAPSII | 45.1 (14.1) | 43.9 (13.6) | 52.3 (14.7) | <0.001 |
| OASIS | 36.8 (8.46) | 36.3 (8.34) | 39.5 (8.70) | <0.001 |
| Charlson | 6.06 (2.87) | 5.92 (2.85) | 6.95 (2.83) | <0.001 |
| APACHEII | 21.9 (7.36) | 21.4 (7.27) | 24.5 (7.34) | <0.001 |
| HR | 91.9 (21.8) | 91.8 (22.0) | 92.6 (20.2) | 0.545 |
| NBPS | 120 (26.1) | 121 (26.0) | 115 (26.0) | 0.001 |
| NBPD | 68.4 (20.2) | 68.8 (20.1) | 66.3 (20.3) | 0.061 |
| RR | 20.1 (6.52) | 20.0 (6.56) | 20.6 (6.23) | 0.203 |
| Spo2 | 96.5 (4.74) | 96.6 (4.67) | 96.1 (5.12) | 0.108 |
| HCT | 31.7 (6.72) | 31.7 (6.65) | 31.4 (7.13) | 0.510 |
| Hb | 10.3 (2.25) | 10.3 (2.23) | 10.2 (2.37) | 0.552 |
| PLT | 204 (116) | 205 (115) | 195 (123) | 0.191 |
| RDW | 16.0 (2.68) | 15.9 (2.63) | 16.6 (2.92) | <0.001 |
| RBC | 3.45 (0.80) | 3.46 (0.79) | 3.39 (0.86) | 0.215 |
| WBC | 13.9 (11.7) | 13.7 (12.1) | 14.7 (8.88) | 0.109 |
| ALB | 2.90 (0.62) | 2.93 (0.61) | 2.69 (0.62) | <0.001 |
| AG | 15.6 (4.91) | 15.5 (4.95) | 16.2 (4.67) | 0.031 |
| TCa | 8.26 (1.03) | 8.28 (1.05) | 8.17 (0.92) | 0.087 |
| Cl | 104 (7.84) | 104 (7.72) | 103 (8.55) | 0.417 |
| Glu | 158 (86.4) | 158 (86.0) | 153 (89.1) | 0.335 |
| K | 4.23 (0.82) | 4.22 (0.81) | 4.31 (0.85) | 0.086 |
| CO2 | 23.9 (6.40) | 24.0 (6.46) | 23.1 (6.00) | 0.018 |
| FCa | 1.11 (0.13) | 1.11 (0.13) | 1.09 (0.12) | 0.019 |
| Lac | 2.45 (2.14) | 2.41 (2.14) | 2.68 (2.14) | 0.060 |
| PCo2 | 42.0 (12.6) | 41.9 (12.5) | 42.2 (12.9) | 0.765 |
| PH | 7.35 (0.11) | 7.35 (0.11) | 7.33 (0.11) | 0.006 |
| Po2 | 127 (103) | 130 (105) | 113 (87.4) | 0.006 |
| INR | 1.67 (1.04) | 1.65 (1.02) | 1.82 (1.15) | 0.019 |
| PT | 18.1 (10.9) | 17.9 (10.6) | 19.7 (12.1) | 0.018 |
| APTT | 40.6 (25.4) | 40.2 (25.6) | 42.8 (24.5) | 0.108 |
| ALT | 152 (680) | 155 (709) | 131 (471) | 0.468 |
| AST | 277 (1310) | 279 (1340) | 264 (1114) | 0.841 |
| TB | 2.15 (5.00) | 1.94 (4.43) | 3.45 (7.47) | 0.001 |
| CRE | 1.81 (1.75) | 1.79 (1.75) | 1.98 (1.74) | 0.084 |
| UREA | 36.1 (29.0) | 34.8 (28.2) | 44.0 (32.3) | <0.001 |
| LDH | 599 (1392) | 569 (1315) | 779 (1783) | 0.062 |
| SA: | 1571 (81.3%) | 1331 (80.3%) | 240 (87.3%) | 0.008 |
| VP: | 1376 (71.2%) | 1151 (69.5%) | 225 (81.8%) | <0.001 |
| GC: | 672 (34.8%) | 561 (33.9%) | 111 (40.4%) | 0.042 |
| Ventilation: | 1792 (92.8%) | 1542 (93.1%) | 250 (90.9%) | 0.251 |
| CRRT: | 281 (14.5%) | 215 (13.0%) | 66 (24.0%) | <0.001 |
| GNRI | 84.2 (9.54) | 84.8 (9.36) | 80.7 (9.86) | <0.001 |
| GNRI group: |  |  |  | <0.001 |
| No | 151 (7.82%) | 139 (8.39%) | 12 (4.36%) |  |
| Low | 260 (13.5%) | 233 (14.1%) | 27 (9.82%) |  |
| Moderate | 682 (35.3%) | 611 (36.9%) | 71 (25.8%) |  |
| High | 839 (43.4%) | 674 (40.7%) | 165 (60.0%) |  |
