# Supplementary material for: The geriatric nutritional risk index predicts short-term mortality in older patients with urosepsis: a retrospective cohort study with external validation
Source: Front Nutr. 2026 Jul 2;13:1793046. doi: 10.3389/fnut.2026.1793046 (PMC13374418; doi:10.3389/fnut.2026.1793046)
Supplement: Supplementary file 5 [file Table_3.docx]

Table S3: Summary descriptives table by groups of ICU dead (External queue verification)

|  | **ALL** | **Survivor** | **No-survivor** | **P value** |
| --- | --- | --- | --- | --- |
|  | ***N=178*** | ***N=149*** | ***N=29*** |  |
| Age | 68.7 (15.8) | 67.7 (16.3) | 73.4 (11.9) | 0.032 |
| Gender: | 78 (43.8%) | 65 (43.6%) | 13 (44.8%) | 1.000 |
| BMI | 26.7 (4.09) | 26.7 (3.98) | 26.5 (4.64) | 0.781 |
| Hyp: | 67 (37.6%) | 61 (40.9%) | 6 (20.7%) | 0.064 |
| CKD: | 58 (32.6%) | 46 (30.9%) | 12 (41.4%) | 0.375 |
| DM: | 70 (39.3%) | 61 (40.9%) | 9 (31.0%) | 0.429 |
| COPD: | 35 (19.7%) | 27 (18.1%) | 8 (27.6%) | 0.359 |
| SOFA | 6.60 (4.03) | 6.26 (3.89) | 8.38 (4.34) | 0.019 |
| APSIII | 56.8 (18.1) | 55.3 (18.0) | 64.4 (16.6) | 0.011 |
| SAPSII | 42.9 (14.0) | 41.3 (12.3) | 51.3 (18.5) | 0.008 |
| OASIS | 36.1 (8.37) | 35.8 (8.31) | 37.6 (8.64) | 0.319 |
| Charlson | 5.43 (2.90) | 5.35 (2.87) | 5.86 (3.06) | 0.409 |
| APACHEII | 22.3 (7.67) | 21.6 (7.22) | 26.0 (8.92) | 0.019 |
| HR | 91.7 (22.2) | 90.6 (22.4) | 97.3 (20.3) | 0.118 |
| NBPS | 119 (25.6) | 120 (25.4) | 112 (25.8) | 0.142 |
| NBPD | 67.7 (19.8) | 68.4 (19.8) | 64.3 (19.4) | 0.307 |
| RR | 19.9 (6.30) | 19.8 (6.13) | 20.1 (7.26) | 0.848 |
| Spo2 | 96.8 (4.96) | 96.7 (4.89) | 96.9 (5.40) | 0.855 |
| HCT | 31.9 (6.60) | 32.2 (6.60) | 30.5 (6.50) | 0.218 |
| Hb | 10.4 (2.16) | 10.5 (2.15) | 9.86 (2.19) | 0.183 |
| PLT | 195 (109) | 197 (109) | 182 (109) | 0.486 |
| RDW | 16.1 (2.70) | 16.0 (2.63) | 16.5 (3.02) | 0.382 |
| RBC | 3.48 (0.74) | 3.53 (0.74) | 3.25 (0.67) | 0.050 |
| WBC | 15.4 (23.9) | 14.2 (19.9) | 21.1 (38.2) | 0.354 |
| ALB | 2.92 (0.52) | 2.98 (0.47) | 2.60 (0.64) | 0.004 |
| AG | 15.7 (4.34) | 15.6 (4.36) | 16.1 (4.28) | 0.628 |
| Cl | 104 (8.23) | 104 (8.44) | 104 (7.10) | 0.718 |
| Glu | 153 (94.2) | 154 (99.7) | 149 (59.3) | 0.701 |
| K | 4.26 (0.85) | 4.31 (0.87) | 4.02 (0.71) | 0.059 |
| CO2 | 24.1 (5.79) | 24.2 (5.95) | 23.8 (4.96) | 0.731 |
| Fca | 1.11 (0.12) | 1.11 (0.13) | 1.08 (0.10) | 0.170 |
| Lac | 2.29 (1.75) | 2.20 (1.80) | 2.76 (1.39) | 0.068 |
| PCo2 | 41.7 (11.7) | 41.2 (11.4) | 44.4 (13.0) | 0.225 |
| PH | 7.36 (0.10) | 7.37 (0.10) | 7.33 (0.10) | 0.095 |
| Po2 | 129 (108) | 132 (108) | 116 (109) | 0.472 |
| INR | 1.75 (1.16) | 1.60 (0.74) | 2.52 (2.22) | 0.035 |
| PT | 18.9 (12.3) | 17.4 (7.51) | 26.8 (24.2) | 0.047 |
| APTT | 41.0 (26.4) | 38.6 (23.8) | 53.5 (34.9) | 0.035 |
| ALT | 172 (639) | 185 (686) | 102 (301) | 0.300 |
| AST | 329 (1610) | 359 (1750) | 173 (395) | 0.250 |
| TB | 1.70 (3.42) | 1.76 (3.67) | 1.40 (1.57) | 0.397 |
| CRE | 1.92 (1.88) | 1.91 (1.97) | 1.94 (1.29) | 0.925 |
| UREA | 38.4 (30.8) | 36.4 (30.0) | 48.3 (33.7) | 0.086 |
| LDH | 856 (2280) | 802 (2253) | 1129 (2439) | 0.508 |
| SA: | 139 (78.1%) | 114 (76.5%) | 25 (86.2%) | 0.363 |
| VP: | 118 (66.3%) | 94 (63.1%) | 24 (82.8%) | 0.066 |
| GNRI | 84.7 (7.93) | 85.7 (7.06) | 79.5 (10.0) | 0.003 |
| GNRI group: |  |  |  | 0.002 |
| No | 9 (5.06%) | 8 (5.37%) | 1 (3.45%) |  |
| Low | 24 (13.5%) | 21 (14.1%) | 3 (10.3%) |  |
| Moderate | 71 (39.9%) | 67 (45.0%) | 4 (13.8%) |  |
| High | 74 (41.6%) | 53 (35.6%) | 21 (72.4%) |  |
